# Dendritic Volume Mesh Reconstruction for STEPS: How Does Mesh Quality Affect Stochastic Reaction-Diffusion Simulation?

**DOI:** 10.1186/1471-2202-14-S1-P95

**Published:** 2013-07-08

**Authors:** Weiliang Chen, Iain Hepburn, Erik De Schutter

**Affiliations:** 1 Computational Neuroscience Unit, Okinawa Institute of Science and Technology, Okinawa 904-0495, Japan; 2 Theoretical Neurobiology, University of Antwerp, B-2610 Antwerp, Belgium

## 

Volume mesh generation is one of the vital procedures in spatial, Gillespie SSA-based [1], reaction-diffusion simulations of dendritic models. Yet over the years few protocols have been established for this procedure due to the highly varied requirements of different research projects. In the attempt of establishing a mesh generation protocol for our spatial SSA simulator, STEPS [2], we investigate the procedures of tetrahedral mesh generation for STEPS and analyze how mesh quality affects simulation results.

STEPS (STochastic Engine for Pathway Simulation) is a GNU-licensed simulation platform that uses an extension of Gillespie's SSA [1] to deal with reactions and diffusion of molecules in 3D tetrahedral mesh reconstructions of neuronal morphology and tissue [2]. In STEPS, the diffusion of molecules is simulated as diffusive fluxes between tetrahedral elements in the mesh, represented by a series of first-order reactions. Tetrahedral meshes used in STEPS simulations are generated in third party mesh generators such as CUBIT [3] and TetGen [4], and then imported via STEPS mesh importing utilities. On one hand, this feature provides maximum flexibility for mesh generation. On the other hand, it requires sufficient understanding and experience of mesh generation and quality control from the user. The case studies presented here can help modelers to avoid various pitfalls during the mesh generation process, which in turn helps to improve the quality of simulation results.

Existing approaches of tetrahedral, dendritic mesh reconstruction fall into two different schemes according to their initial geometry representations. The common approach starts from a computational Constructive Solid Geometry (CSG) representation of the modeling subject, formed by Boolean operations on standard geometry primitives such as cubes, cylinders and spheres. The second approach begins with a contour tracing representation from experimental Electron Microscopic images. Meshing algorithms such as Delaunay triangulation and tetrahedralization [5] can then be applied to both representations to create tetrahedral meshes for the simulation. Although the first approach is widely used in present neuroscience research due to its simplicity, the lack of morphological accuracy significantly limits its application. The second approach is more advanced, producing meshes with high morphological accuracy, whilst its quality control can be a great challenge since experimental data acquisition can often not be repeated.

In our investigation, we apply meshes that are generated from both approaches to STEPS simulations. Different mesh quality is achieved by varying the parameter settings of the mesh generator. Multiple instances of the simulation are executed for each mesh and the results are averaged. For simple morphologies where analytical solutions exist, we compare the simulation results with the analytical solutions, or else results of different quality meshes for the same morphology are compared. Based on these comparisons, we aim to reveal how SSA simulation is affected by mesh quality, and to draw guidelines of mesh generation for STEPS.
